# Tumorigenic potential is restored during differentiation in fusion-reprogrammed cancer cells

**DOI:** 10.1038/cddis.2016.189

**Published:** 2016-07-28

**Authors:** J Yao, L Zhang, L Hu, B Guo, X Hu, U Borjigin, Z Wei, Y Chen, M Lv, J T Y Lau, X Wang, G Li, Y-P Hu

**Affiliations:** 1 Department of Cell Biology, Center for Stem Cells and Medicine, Second Military Medical University, Shanghai 200433, People's Republic of China; 2 Department of Cell Biology and Genetics, School of Basic Medical Sciences, Xi'an Jiaotong University Health Science Center, Xian 710061, People's Republic of China; 3 Key Laboratory of Molecular and Cell Biology, Shanghai Institutes for Biological Sciences, Chinese Academy of Sciences, Shanghai 200031, People's Republic of China; 4 Basic Medical College, Shanxi University of Traditional Chinese Medicine, Shanxi 030024, People's Republic of China; 5 Key Laboratory of National Education Ministry for Mammalian Reproductive Biology and Biotechnology, Inner Mongolia University, Huhhot 010021, People's Republic of China; 6 Pearl Laboratory Animal Science and Technology Co. Ltd, Guangzhou, People's Republic of China; 7 Department of Molecular and Cellular Biology, Roswell Park Cancer Institute, Buffalo, NY 14263, USA; 8 Hepatoscience Inc., Sunnyvale, CA, USA

## Abstract

Detailed understanding of the mechanistic steps underlying tumor initiation and malignant progression is critical for insights of potentially novel therapeutic modalities. Cellular reprogramming is an approach of particular interest because it can provide a means to reset the differentiation state of the cancer cells and to revert these cells to a state of non-malignancy. Here, we investigated the relationship between cellular differentiation and malignant progression by the fusion of four independent mouse cancer cell lines from different tissues, each with differing developmental potentials, to pluripotent mouse embryonic stem (ES) cells. Fusion was accompanied by loss of differentiated properties of the four parental cancer cell lines and concomitant emergence of pluripotency, demonstrating the feasibility to reprogram the malignant and differentiative properties of cancer cells. However, the original malignant and differentiative phenotypes re-emerge upon withdrawal of the fused cells from the embryonic environment in which they were maintained. cDNA array analysis of the malignant hepatoma progression implicated a role for Foxa1, and silencing Foxa1 prevented the re-emergence of malignant and differentiation-associated gene expression. Our findings support the hypothesis that tumor progression results from deregulation of stem cells, and our approach provides a strategy to analyze possible mechanisms in the cancer initiation.

Investigations into cancer formation are most often focused on the accumulation of specific genetic and epigenetic alterations that alter the expression of the oncogenes and tumor suppressors regulating cell cycle, apoptosis, DNA repair, cell adhesion and signaling.^1, 2, 3^ Less often considered, the tumorigenic process can also be regarded from a standpoint of a dynamic relationship between malignant progression and cellular differentiation.^4^ During the course of development, normal stem cells differentiate into specific types of cells by exchanging and interpreting signaling molecules with the surrounding microenvironment. Accumulating evidence indicates that cancer cells may also release and receive cues from the surroundings that contribute to malignant progression.^5^ However, how tumor cell–niche interactions drive malignancy remains a critical gap in our overall understanding of the cancer process, and understanding this process has significant potential in providing new prognosis strategy for therapeutic intervention at early stages of cancer development.

Reprogramming can alter differentiation properties of adult cells, and this approach may be exploitable to reverse the malignant programming in cancer cells.^6^ Published reports documented the use of nuclear transfer by implanting the nuclei of mouse melanoma,^7, 8^ embryonic carcinoma^8^ and medulloblastoma^9^ into mouse oocytes. Although the nuclear transferred cells regained pluripotent potential, the malignant properties remained, indicating incomplete reprogramming in reproductive and therapeutic cloning with this approach.^10, 11^ Separately, defined factors OSMK (Oct4, Sox2, c-Myc and Klf4) were tested for the ability to reprogram both solid and liquid malignant tumors including chronic myeloid leukemia,^12, 13^ gastrointestinal cancer,^14^ melanoma^15^ and sarcoma cells.^16, 17^ Using the OSMK approach, late-stage cancer cells could revert back to an earlier state, bolstering enthusiasm for the discovery of new insights in cancer initiation and progression. However, OSKM-reprogrammed cells had limited pluripotency and altered tumorigenic potential during re-dedifferentiation. Moreover, the OSKM approach to promote pluripotency was effective only on a limited subset of cancer types.^18^ The shortcomings of OSMK may be due to the presence of oncongenic factors (c-Myc and Klf4) or to the intrinsic defects of the strategy.^19, 20^ Most importantly, these shortcomings hinder the use of OSKM approach to investigate tumor progression in reprogrammed cancer cells.

ES cell-induced fusion provides a more efficient and effective reprogramming strategy to test the reversibility of tumorigenic potential. In previous studies using normal adult cells, the normal cell fusion hybrids exhibited epigenetic characteristics similar to ES cells, such as reactivation of histone modifications and a DNA hypomethylation state within the *Oct4* promoter.^21, 22, 23, 24, 25, 26, 27, 28, 29^ We generated a fusion hybrid of mouse hepatoma cells and mouse embryonic stem (ES) cells previously.^30^ The resultant ES-Hepa hybrids forfeited tumorigenic properties, but the forfeiture was reversible and tumorigenic properties re-emerge upon removal of the cells from embryonic environments. We observed that H3K27 trimethylation, which was independent of H3K9 dimethylation, was an early event in the silencing of *p16*^*INK4a*^ during re-emergence of the tumorigenic profile, a finding that was supported by a number of other groups studying the progression mechanisms of hepatocellular carcinoma (HCC).^30, 31, 32^ These previous research highlighted the remarkable developmental plasticity of HCC during cancer progression and engendered two important questions. First, is developmental plasticity a ubiquitous phenomenon in all cancer progression? Second, how does lineage specification relate to cancer progression? While the reprogramming approach holds significant promise for future cancer therapies, current data also caution that partial or incomplete reprogramming can lead to a worse outcome by inducing more invasive phenotypes.^33^ Clearly, much critical knowledge remains to be learned regarding the association between the differentiation and tumorigenic phenotypes in cellular reprogramming and the molecular events driving these events.

Here, we present data showing that four cancer cell lines, each endowed with distinct lineage-differentiated characteristics, were reprogrammed by fusion with ES cells to yield ES-cancer hybrids with characteristics similar to pluripotent ES cells with diminished tumorigenic gene expression patterns. We observed that the sustainability of pluripotent ES characteristics was dependent on environmental cues. Removal of cells from embryonic culturing conditions resulted in the re-emergence of the original differentiated and tumorigenic gene expression patterns. cDNA array and RT-PCR data in malignant hepatoma progression discovered Foxa1 as a potential checkpoint regulator, affecting a network of 224 genes that might be participants for the differentiation and malignant progression. Silencing of Foxa1 inhibited the re-emergence of the malignant and differentiated pattern of gene expression. Our observations suggest that cell fusion-mediated reprogramming is a novel and potentially useful strategy for the identification and testing of molecules involved in cancer initiation and progression, with exploitable modalities for new cancer therapies.

## Results

### Generation of ES-fusion hybrids of embryonic cancer and adult cancer cells

**Figure 1:**
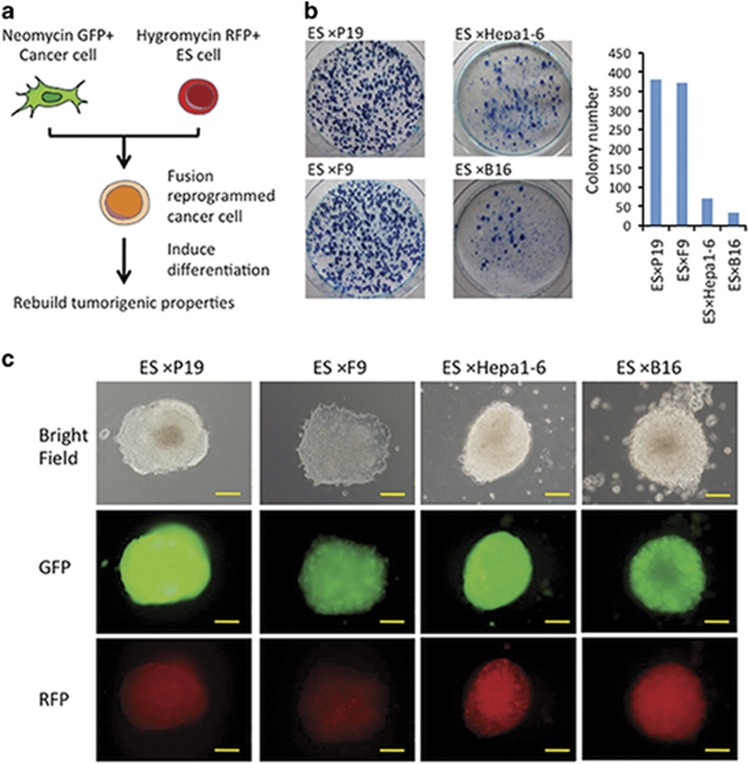
The formation of hybrid colonies from ES cell and cancer cell fusions. (**a**) The strategy was to generate hybrids by mouse ES cell and cancer cell fusions. Cancer cells with distinct differentiation potentials were fused with ES cells to generate stable hybrid cells. Existing cancer cells and ES cells were stably transfected with independent drug-resistant and fluorescent markers. Fused cells were generated in the presence of polyethylene glycol (PEG) and grown under standard conditions in the presence of antibiotics to select for double fluorescence-positive and double resistance-positive cell hybrids. After differentiation, the tumorigenic properties were rebuilt. (**b**) Plates containing hybrid colonies from PEG fusions of ES cells and cancer cells are shown on the left. The colonies of each plate were counted, and the numbers are compared on the right. (**c**) Morphological characters of the EP, EF, EHe and EB cell lines. Microscopic images of bright field (top), green fluorescence (middle) and red fluorescence (bottom) are shown. Scale bar: 100 *μ*m

To investigate the association between the reprogramming potential of cancer cells to their differentiation status, cancer cells of different layers and development levels were fused to ES cells that constitutively expressed RFP and hygromycin resistance, as described previously.^34^ Four cancer cell lines were used, the GFP-expressing and neomycin-resistant embryonic carcinoma cells (P19 and F9), and the adult cancer cells, Hepa1-6 and B16 (Supplementary Table S1). The cell hybrids were selected in hygromycin and neomycin for 2–3 weeks, and the resultant colonies counted to evaluate the fusion efficiency (Figure 1a). Analysis of chromosome spreads indicated that almost every hybrid line had a near-tetraploid chromosome complement of 80 (Supplementary Figure S1A). Propidium iodide (PI) analysis confirmed that the hybrids contained nearly 4*n* DNA content (Supplementary Figure S1B). Six colonies of each hybrid were picked for further analysis. All double-positive colonies that were picked had similar phenotypes and growth rates, which remained consistent even after 65 passages.

We also noted that ES-fusion hybrids of adult cancer cells (Hepa1-6 and B16) were generated at 5–10-fold less efficiently than the ES fusions of embryonic carcinoma cells (P19 and F9) (Figure 1b). Although the colonies of ES-P19 (EP) and ES-F9 (EF) hybrids had similar colony shape to those of ES-adult cancer cells (Figure 1c), colonies of both ES-embryonic carcinoma hybrids appeared much earlier than those of the ES-adult cancer cell hybrids, 10 and 16 days, respectively (data not shown), suggesting that embryonic carcinoma cells were reprogrammed more readily than adult cancer cells.

### Pluripotency phenotype is dominant over malignant phenotypes in ES-cancer hybrids

**Figure 2:**
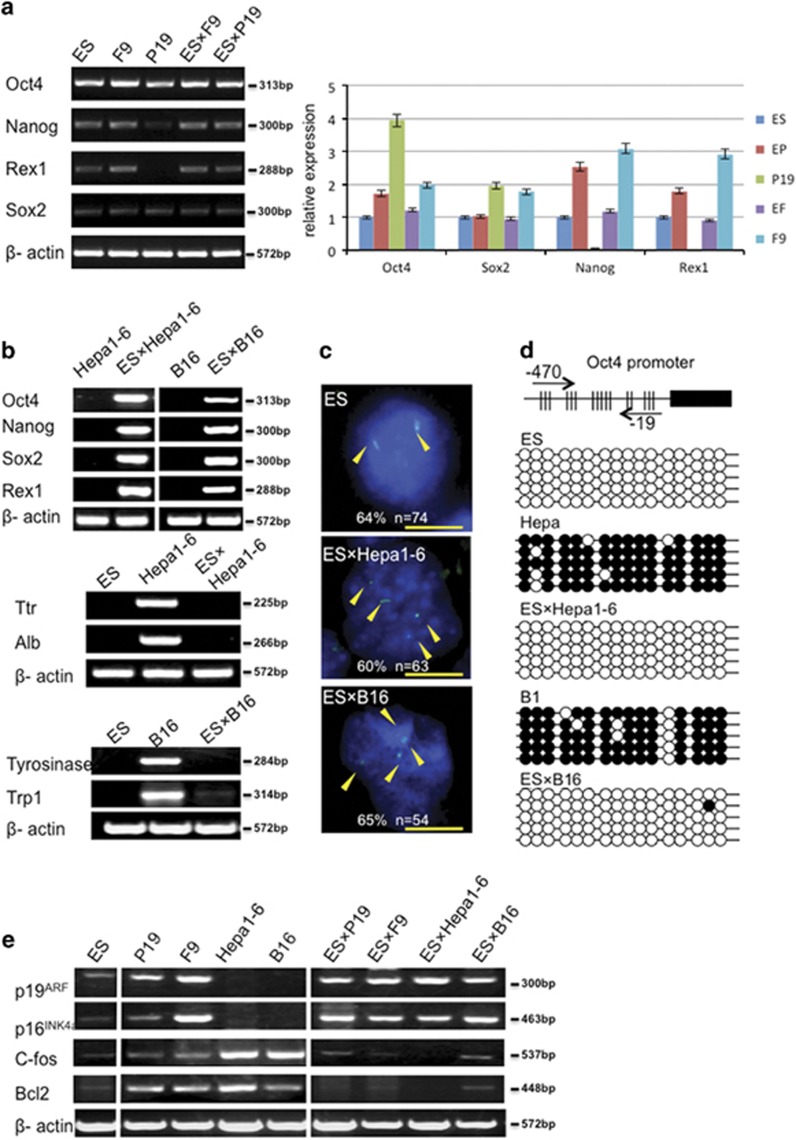
Gene expression analysis in ES cells, cancer cells and ES-cancer cell hybrids. (**a**) Pluripotent gene expression in ES-embryonic carcinoma cells were analyzed by reverse transcription-PCR (RT-PCR) and real-time PCR. Error bars, S.E. of the average values. (**b**) Pluripotent gene expression (top) and tissue-specific gene expression (bottom) in EHe and EB lines were analyzed by RT-PCR. (**c**) RNA-FISH staining for Oct4 in EHe and EB hybrid cells. Yellow arrowheads indicate Oct4 RNA nascent transcripts. The percentage of cells showing the expected allelic expression is indicated. Representative images show sites of Oct4 transcription (green) merged with 4′, 6-diamidino-2-phenylindole (DAPI) in ES, Hepa1-6, B16, EHe and EB hybrid cells. Scale bar, 50 *μ*m. (**d**) Bisulfite genomic sequencing of the promoter regions of Oct4 in ES-cancer hybrid cells, ES cells and cancer cells. Open circles indicate unmethylated CpG dinucleotides, whereas closed circles indicate methylated CpGs. (**e**) Cancer-related gene expression in ES cells, cancer cells and ESC-cancer cell hybrids were analyzed by RT-PCR

The expression of tissue-specific genes and pluripotent genes were examined in the ES-cancer cell hybrids. Similar to the parental ES cells, the pluripotency genes *Oct4*, *Nanog*, *Sox2* and *Rex1* were also expressed in the hybrids (Figure 2a). Among the ES-embryonic carcinoma hybrids, the pluripotency gene expression pattern of both EP and EF hybrids was more similar to the parental ES than to their respective parental embryonic carcinomas (Figure 2a). For the ES-adult cancer hybrids, ES-Hepa and ES-B16 (EB), both hybrids had extremely elevated expression of pluripotency genes, and these genes were not expressed at all in the parent adult cancer cell lines Hepa1-6 and B16 (Figure 2b). Moreover, expression of the liver-specific (*Ttr* and *Alb*) and melanin-specific genes (*tyrosinase* and *Trp-1*), abundantly expressed in the parent adult cancer cells, decreased significantly in the hybrids (Figure 2b).

To document further the reactivation of the silenced *Oct4* in cancer cells upon hybrid formation, we measured the allelic expression of *Oct4* by RNA-fluorescence *in situ* hybridization (RNA-FISH). The four dots per nuclei signals were obvious in the ES-cancer hybrids, but absent in the parental cancer cells, demonstrating reactivation of the silenced *Oct4* upon fusion to ES cells (Figure 2c). Moreover, CpG islands in the promoter region of *Oct4* gene were unmethylated in the transcriptionally active *Oct4* of the ES and ES-cancer hybrids, but methylated in the silenced *Oct4* of the adult cancer cells, further supporting that the reactivation of the silenced pluripotent gene (Figure 2d). Thus, all four distinct cancer cells types from four different origins could be reprogrammed to the pluripotent status by fusion with ES cells.

To assess if the pattern of tumor gene expression in the parental cancer cells could be returned to a comparatively benign states, we analysed the expression of two tumor suppressors (*p19*^*ARF*^ *and p16*^*INK4a*^) and two oncogenes (*Bcl2 and C-fos*). For the tumor suppressor genes, ES-fusion brought about normalization of *p19*^*ARF*^ *and p16*^*INK4a*^ levels, from undetectable levels in the adult cancer cells (Hep1-6 and B16) or from comparatively high levels in the embryonic carcinomas (P19 and F9) (Figure 2e). For the oncogenes, ES-fusion virtually extinguished the expression of both *Bcl2 and C-fos* in the adult cancer cells (Hepa1-6 and B16), as well as in the embryonic carcinomas (P19 and F9) (Figure 2e). Taken together, these observations point to reprogramming of the cancer cell gene expression patterns to the pluripotent status upon fusion to the ES cells.

### Differentiation accompanies malignant progression in ES-cancer cell hybrids

**Figure 3:**
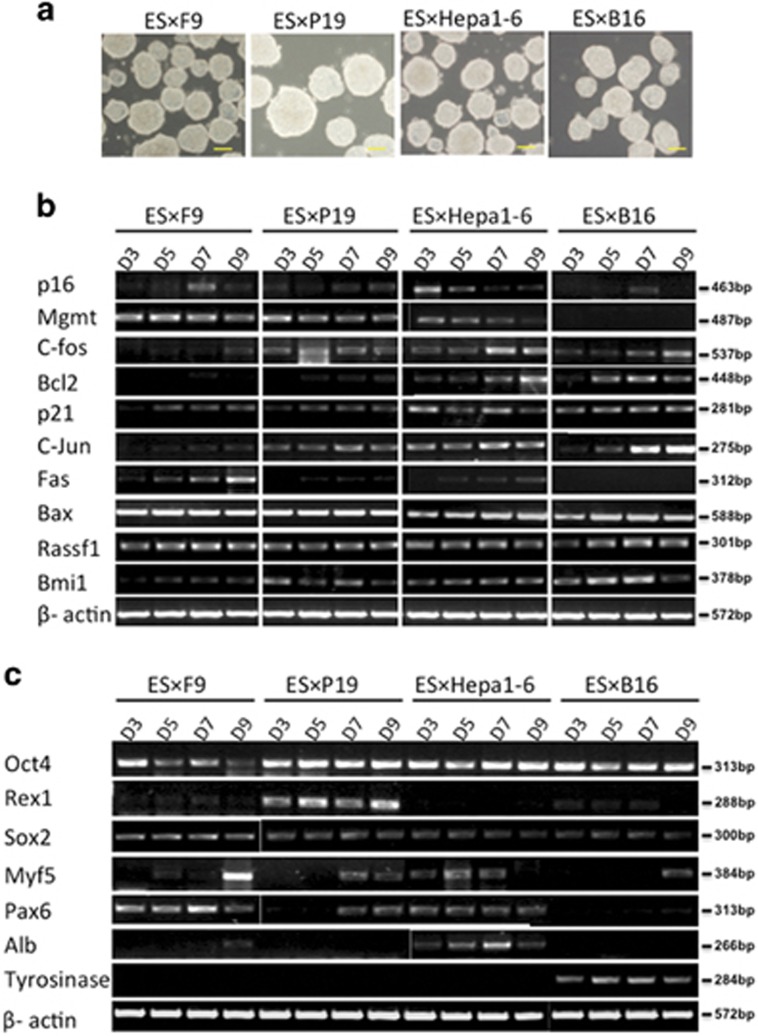
Induce ES cells and hybrids to differentiate *in vitro*. (**a**) Morphology of representative day 5 embryoid bodies derived from ES-cancer cell hybrids. Scale bar, 100 *μ*m. (**b**) Reverse transcription-PCR (RT-PCR) analysis of tumor-related gene expression in ES-cancer hybrid cells over the indicated number of days. *β*-Actin was used as a ubiquitously expressed control. (**c**) RT-PCR analysis of tissue-specific gene and pluripotent gene expression in ES-cancer hybrid cells over the indicated number of days. *β*-Actin was used as a ubiquitously expressed control

ES cell differentiation is controlled by epigenetic modulations responding to autocrine and paracrine delivery of signaling molecules. Malfunctioning epigenetic controls can result in improper cellular responses to environmental cues, leading to abnormal differentiation programs. First, to examine the relationship between tumorigenic potential and differentiation status, ES and ES-cancer hybrids were differentiated *in vitro* into embryonic bodies for 3, 5, 7 and 9 days (Figure 3a). As shown in Figure 3b, tumor suppressor *P16* ^*INK4a*^ and *Mgmt* expression were downregulated gradually upon differentiation, whereas expressions of oncogenes such as *C-fos*, *Bcl2*, *p21*, *C-jun*, *Fas* and *Bmi1* were upregulated (Figure 3b). These results suggest that induction of the ES-cancer cell hybrids to differentiation restores tumor gene expression. The pluripotent gene panel, including *Oct4*, *Sox2* and *Rex1*, was also examined (Figure 3c). Upon differentiation, *Oct4* expression remained high when compared with the other two pluripotent-related genes, suggesting that ES-cancer hybrids retained vestiges of a ‘stemness state' (Figure 3c), consistent with the previous report that *Oct4* was involved in the expression pattern of ‘cancer stem cells' and its expression is correlated with advanced tumor grade.^35^ Tissue-specific genes were also examined, including the hepatic marker *Alb*, *the* melanin marker *tyrosinase* and ectoderm and mesoderm markers by RT-PCR. The results support the idea that differentiation potential was retained in the ES-cancer hybrids. For the adult cancer cell hybrids, ES-Hepa and EB, *in vitro* differentiation led to the re-emergence of cancer cell tissue-specific expression phenotype (Figure 3c). The precise expression patterns of each ES-cancer differ may vary depending on the different genetic origins of the individual parental cancer lines. Nevertheless, these results showed the association of tumor-related gene expression with the differentiation status of the cancer cells.

### Differentiation-related tumorigenic transcriptome in ES-cancer hybrids

**Figure 4:**
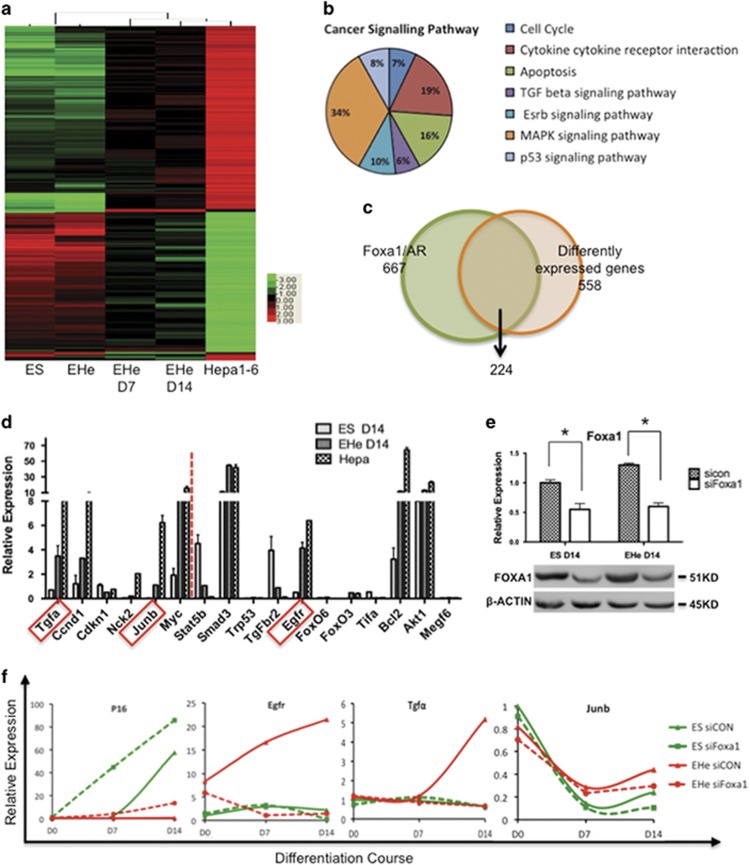
Identification and verification of differentiation-related tumorigenic genes from Foxa1/AR targets. (**a**) Analysis of gene expression by cDNA microarray assay. Expression profiles were clustered by a Pearson's correlation analysis. Expression levels are depicted in color. (**b**) Functional pathway analysis of 558 differential gene expressions (fold change >3, *P*<0.01, *t*-test) in undifferentiated and differentiated ES-hepatoma hybrids. (**c**) Intersect analysis of 667 potential cancer-promoting genes that are targeted by Foxa1/AR and 558 differentially expressed genes from the array. (**d**) Quantitative PCR (qPCR) analysis of transcripts in hepatoma cells, ES and EHe hybrids differentiated at day 14. Genes on the left side of the red dash are the ones being bound at the promoter region, whereas genes on the left side are the ones being bound at the intron enhancer region. *Tgfa*, *Junb* and *Egfr* are the genes being bound at both of the regions. Transcripts of ES cells at day 0 were normalized to 1. Error bars, S.E. of the average values. (**e**) The decreased expression level of Foxa1 by siRNA was verified by qPCR and western blot. Transcripts of control siRNA (sicon)-transfected ES cells at differentiation day 14 was normalized as 1. Error bars, S.E. of the average values. *P<0.05. (**f**) Dynamic expression level of oncogenic Foxa1-binding genes (*Tgfa*, *Egfr* and *c-Jun*) and non foxa1-binding gene *p16*^*INK4a*^ from days 0 to 14 in sicon and Foxa1 silencing group (siFoxa1). Transcript levels at day 0 were normalized to 1

Global expression profiles among the ES cells, Hepa1-6, ES-Hepa hybrids and differentiated ES-Hepa hybrids at days 7 and 14 postinduction were examined comparatively. Pearson's correlation analysis showed undifferentiated ES-Hepa hybrids clustered with ES cells, but separated from the differentiated ES-Hepa hybrids and Hepa1-6 cells (Figure 4a). At the same time, differentiated ES-Hepa hybrids clustered with Hepa1-6 cells. When the ES-Hepa hybrids were kept in an undifferentiated state, the pattern of tumorigenic gene expression was similar to that of the ES cells. When induced to differentiate, the ES-Hepa hybrids acquired gene expression profiles with strong similarity to that of Hepa1-6 cells (Supplementary Table S2 and Supplementary Figure S2). Microarray data revealed numerous changes in the expression of cancer-related genes upon differentiation of the ES-Hepa hybrids, where 643 out of 29 153 annotated genes were found to be upregulated in differentiated ES-Hepa hybrids, and 801 genes were downregulated (fold change >3, *P*<0.01, *t*-test) upon differentiation. To identify which among the differently expressed genes contribute to tumorigenesis, 558 tumorigenesis-related genes were classified into seven groupings corresponding to pathways in cancer: cell cycle, apoptosis, cytokine cytokine receptor interaction signaling pathway, MAPK signaling pathway, TGF*β* signaling pathway, p53 signaling pathway and Esrb signaling pathway (Figure 4b, Supplementary Table S2 and Supplementary Figure S2). When the ES-Hepa hybrids were induced to differentiate *in vitro*, the genes in these pathways generally showed a re-emergence of the parental Hepa1-6 patterns of expression, but in general they did not return to the same expression levels as in the Hepa1-6 cells after 7 and 14 days (Supplementary Table S2 and Supplementary Figure S2). Moreover, the individual ES-Hepa hybrids expressed relatively variable levels of expression of these genes, even in the undifferentiated state. Therefore, at days 7 and 14 of differentiation, the gene expression pattern may reflect an intermediate stage to the final tumorigenic end point represented by the Hepa1-6 cells. Despite these minor variations, an association of the cancerous pattern of gene expression in the ES-Hepa hybrids with differentiation status was demonstrated by the transcriptome analysis.

Foxa1 has long been recognized as a factor for the formation of foregut definitive endoderm (DE) cells and further liver bud development by decompacting chromatin and repositioning nucleosomes.^36, 37^ Recently, Foxa1 has been found to exert a dominant role in male HCC development by recruiting androgen receptor (AR) to its binding sites.^38^ To understand the regulatory function of Foxa1 in activation of the tumorigenesis, 667 Foxa1/AR dual-associated targets^38^ were intersected with the 558 differently expressed genes in the differentiated ES-Hepa hybrids group. We found that 40% (224/558) of the differently expressed genes were targets of Foxa1/AR binding genes (Figure 4c), indicating enhanced regulation of Foxa1 during carcinogenesis. Among the 224 genes, 6 genes that bound at the promoter region and 11 genes bound at the transcriptional enhancer region exhibited malignant expression levels after being differentiated as verified by qRT-PCR (Figure 4d). To determine whether Foxa1-binding affects gene expression of the targets identified by ChIP-Seq, we knocked down Foxa1 in ES and ES-Hepa hybrid cells by siRNA. As shown in Figure 4e, Foxa1 expression at the RNA and protein levels was decreased in both the differentiated ES and ES-Hepa hybrid cells at day 14. We focused on three oncogenes (Tgfa, Junb and Egfr) that bound Fox1 at both the promoter region and the intron enhancer region, and one tumor suppressor gene (*p16*^*Ink4a*^) that is not directly related to Foxa1. Knockdown of Foxa1 resulted in a significant decrease in the expression of Tgfa and Egfr and an increase in the expression of *p16*^*Ink4a*^, while the expression of Junb remained unchanged. Taken together, these findings support the idea that the identified Foxa1-binding sites are participants in malignant progression in our cancer initiation model of hybrid cell differentiation.

### *In vivo* tumorigenic potency of ES-cancer hybrids re-emerges after transplantation

**Figure 5:**
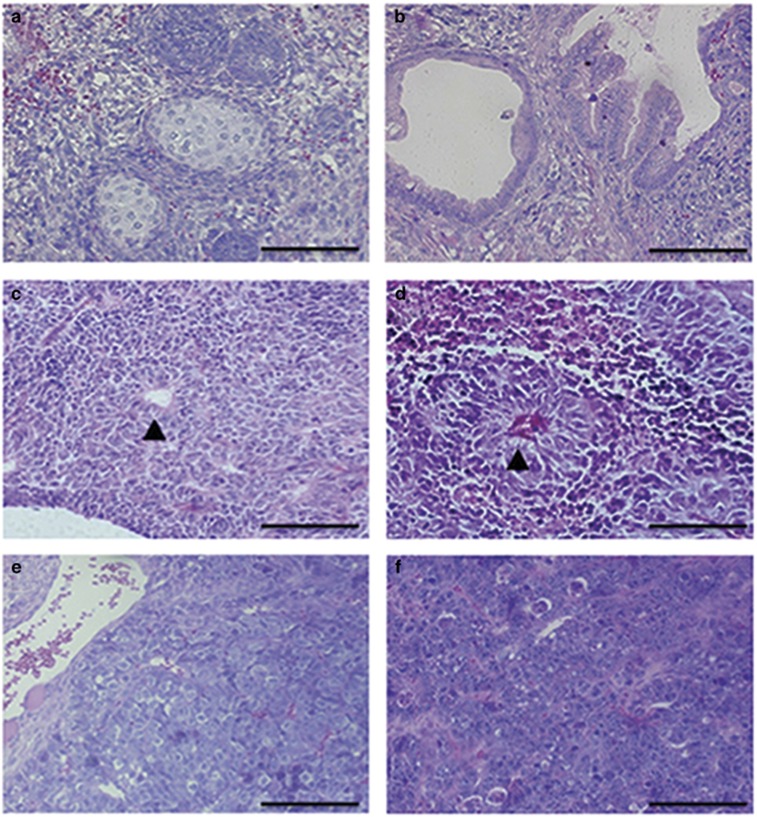

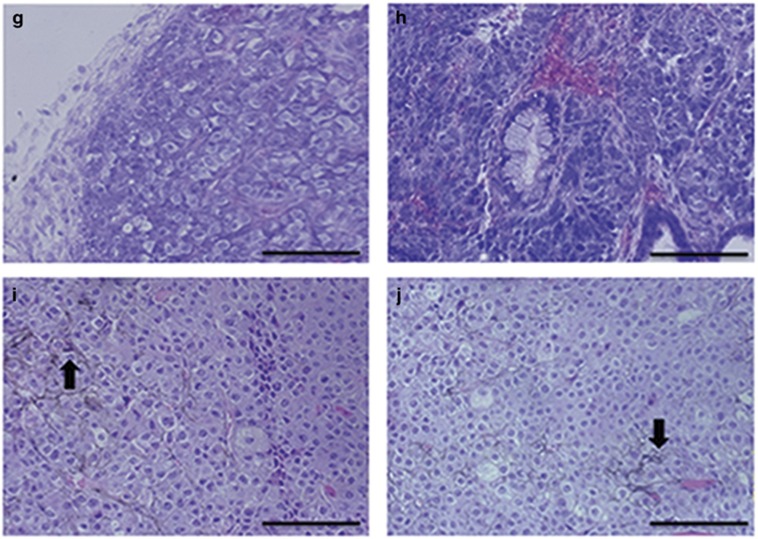
Teratoma analysis of hybrid cells and corresponding parental cell lines. ES cells, cancer cell lines and ES-cancer cell hybrids were injected subcutaneously into nude mice. Representative haematoxylin- and eosin-stained sections of tumors from (**a**) ES cells; (**b**) ES-lymphocyte cell hybrids established broad range of mature tissue types; (**c**) P19, (**d**) EP hybrids can spontaneously differentiated into immature neuroepithelium marked by rosette formation (▴); (**e**) F9 and (**f**) EF hybrids established undifferentiated tissue types in the teratoma; (**g**) Hepa1-6-derived tumor showed a hepatocarcinoma tissue type; and (**h**) in tumors derived from EHe hybrids, >80% of the areas showed undifferentiated malignant cell types with enlarged nuclei and less cytoplasm; in tumors derived from B16 (**i**) and 70% area of EB hybrids (**j**), undifferentiated cells with hyperchromic nuclei and melanin granule (**↑**) were observed. Scale bar, 1000 *μ*m

To exclude the possibility that increased tumorigenic potential might be acquired simply by fusion to ES cells, a normal lymphocyte was also fused to ES. ES cells, ES-lymphocyte and ES-cancer cell hybrids were injected subcutaneously into immune-deficient nude mice and teratoma formation was monitored. The teratoma growth rate of ES-lymphocyte cell hybrids was similar to that of the native ES cells (4 weeks) (Table 1). On the other hand, the teratomas derived from ES-cancer cell hybrids were distinct from those of ES cells and ES-lymphocyte hybrids by both rate of tumor formation and the percentage of immature tissues (Table 1). First, the rate of tumor formation (2 weeks) of all ES-embryonal carcinoma (EC) cell lines was quite similar to that of the parental EC lines F9 and P19, which was much faster than the rate of ES cells and ES-lymphocyte cell hybrids (Table 1). The rate of the formation of visible teratomas was also much faster (2 weeks) for the ES-Hepa and EB hybrids compared with the ES cells (4 weeks) and the adult cancers (4–5 weeks), which showed a tumor growth rate similar to EC and ES-EC cell hybrids (2 weeks) (Table 1). Second, the hematoxylin and eosin staining of the teratomas showed that EP cells had the same limited differentiation ability to form immature neuroepithelium marked by rosette formation as P19 cells (Figures 5c and d). Similar results were also found in the histology analysis of tumors derived from EF hybrids, and teratoma tissues from EF were similar to those derived from the parental F9 cells, which were totally undifferentiated cells with deformed, enlarged nuclei and less cytoplasm (Figures 5e and f). The tumors derived from ES-Hepa hybrids exhibited enhanced differentiation ability because all three layers – including cartilage, adipose tissue, glial cells and mature epithelial cells – were observed in the teratomas, unlike the homogeneous hepatoma tumors of Hepa1-6 (Figure 5g). However, the tumors derived from ES-Hepa cells were also dissimilar to teratomas derived from ES cells and ES-lymphocyte hybrids (Figures 5a and b), as exemplified by the much higher percentage area of undifferentiated cells in tumors derived from ES-Hepa hybrids, compared with ES cell-derived tumors (80% and 20%, respectively), and the presense of obvious cancer nests in the ES-Hepa-derived tumors (Figure 5h). Furthermore, spontaneously differentiated tumors from EB hybrids contained three germ layers, but >70% of the tumor's area established typical melanoma tissue types of undifferentiated cells with hyperchromic nuclei and melanin granules (Figures 5i and j). These observations demonstrate that ES-cancer cells are capable of differentiating, and differentiation is associated with the restoration of tumorigenic potential.

## Discussion

In this study, two adult cancer lines (mouse Hepa1-6 and B16) and two EC lines (mouse P19 and F9) were fused with mouse ES cells to investigate how tumorigenic potential of cancer cells can be influenced by differentiation status. These cancer cell lines were selected to examine several different aspects driving tumorigenic potential. First, different epigenetic regulations exist in embryonal and adult cancer cells. In EC cells, for example, the promoters of some tumor suppressor genes are epigenetically repressed to lesser degrees than in adult cancer cells.^6^ Therefore, tumorigenic potential of the EC cells may be more likely to be lost completely and cannot re-emerge during the subsequent differentiation course. Second, hepatoma cells and melanoma cells are typical malignant tumors that endanger lives of millions of people. Different from EC cells, these hepatoma and melanoma cells have genetic, in addition to epigenetic, irregularities. Therefore, comparative analysis of the molecular pathways of tumorigenesis of the two cancer types can provide insight into prognosis strategies. In the present research, these cancer cell lines were fused with ES cells, and all ES-cancer cell hybrids showed similar pluripotent gene expression levels and cell growth rates. However, colony formation time of ES-EC cell hybrids was shorter than that of ES-adult cancer cell hybrids, and the number of the double-resistant and dual-fluorescent colonies of ES-EC cell hybrids was much higher compared with that of the ES-adult cancer cell hybrids. This observation supports the findings of previous studies which demonstrated that the differentiation status of somatic cells was a critical parameter affecting the fusion rate.^39^

Previous studies have shown that ECs and melanomas can be reprogrammed by nuclear transfer. Tumorigenic potential, although initially silent, can re-emerge later upon induction of the nuclear transferred ES cells to differentiate.^7, 8^ In this study, we observed the similar outcomes. The phenomenon of reacquisition of tumorigenic phenotypes may be the result of bidirectional cellular communication with the surrounding microenvironment. This idea is supported by recent findings that embryonic microenvironments can alter aggressive cancer malignancy by reducing tumorigenesis and metastasis.^40, 41, 42, 43^ Therefore, one can suggest that alterations of the microenvironment, such as inflammation, may have an important role in tumorigenesis.

Our observations also support the claim that the differentiation status of cancer cells is important in tumor development. We have exploited the relationship between cancer cells and their microenvironment, within which the differentiation status of the cancer cells is controlled by epigenetic modulations in response to autocrine and paracrine delivery of signaling molecules. Our data showed that bidirectional communication with the microenvironment is related to progression and development of the cancer cell. These findings have promising implications, suggesting that an embryonic microenvironment can silence tumorigenic phenotypes, while a differentiation environment pushes reacquisition of the ‘tumorigenic program'.

More important, the reprogrammed adult cancer cells preferred to recapitulate the original linage and malignant program during the self-differentiation course, suggesting that factors that are responsible for linage development might have a relationship with the linage-related tumorigenic program.^44^ During liver development, *Foxa1/2* transcription factors establish competence by opening compacted chromatin structures within liver-specific target genes and the onset of hepatogenesis.^36, 37^ In cancer, *Foxa1* is amplified and overexpressed in esophageal,^45^ lung,^46^ prostate^47^ and breast cancers,^48^ suggesting an oncogenic potential in the epithelial cancers. More interestingly, *Foxa1* and *Foxa2* cooperate with AR to promote diethylnitrosamine-induced hepatocarcinogenesis in male mice.^38^ In the present study, we tested Foxa1 tumorigenic potential in ES-Hepa hybrid differentiation, and our data suggest participation of Foxa1 in the tumorigenic program by contributing to cancer progression. Knockdown of Foxa1 resulted in increased expression of tumor suppressor genes and concomitant decreased expression of oncogenes. Further investigations into the analysis of molecular interaction within the overall ‘tumorigenic program', as performed for the *Foxa1* shown in our data, should allow identification and testing of additional potential candidate molecules involved in cancer development and progression.

## Materials and Methods

### Cell lines

E14 ES cells were cultured in Glasgow minimum essential medium (GMEM) (Gibco-BRL, Grand Island, NY, USA) supplemented with 10% knockout serum replacement (KSR; Gibco), 1% fetal bovine serum (Hyclone, Logan, UT, USA), 1 × penicillin/streptomycin/glutamine, 1 × nonessential amino acids (Gibco-BRL), 0.1 mM 2-mercaptoethanol, 1 mM sodium pyruvate and 1000 U/ml leukemia inhibitory factor (ESGRO; Chemicon, Temecula, CA, USA). EC cell P19 and F9 cell lines (passages unknown) were grown on gelatin-coated (0.1% in phosphate-buffered saline (PBS)) dishes in standard EC cell medium, which is high-glucose Dulbecco's modified Eagle's medium (DMEM) (Gibco-BRL, Gaithersburg, MD, USA) supplemented with 10% fetal calf serum (FCS; Gibco-BRL), 1% penicillin/streptomycin/glutamine and 1% nonessential amino acids (Gibco-BRL). The mouse hepatoma cell line Hepa1-6 and the melanoma B16 line were cultured in high-glucose DMEM (Gibco-BRL) containing 10% FCS (Gibco-BRL) and 1% penicillin/streptomycin/glutamine. Thymocytes collected from 6–8-week-old GFP transgenic mice were passed through an 18-gauge needle several times to create single-cell suspensions.

### Generation of transgenic cell lines

To generate transgenic mouse ES cell lines, cells were transduced with replication-incompetent lentiviral vector with a hygromycin resistance gene and an RFP gene, driving by an EF-1*α* promoter. At 48 h after viral transduction, hygromycin B (Invitrogen, Shanghai, China) was added to the medium at a concentration of 25 *μ*g/ml for 2 weeks. Following drug selection, individual colonies were picked and expanded into lines. To generate drug-resistant cancer cells, F9, P19, B16 and Hepa1-6 cells were transfected with a neomycin resistance gene and a GFP gene using a transient transfection kit. At 48 h after viral transduction, neomycin (Gibco) was added to the culture medium at a concentration of 100 *μ*g/ml, and individual colonies were picked at day 14 and expanded into lines. The EF-1*α* promoter system was chosen to establish a robust, constitutive and long-termed lentiviral expression system.^49, 50, 51^ All established hybrid lines remained double fluorescence-positive for >65 passages *in vitro*.

### Cell fusion

For PEG fusions, cells of each type (generally 5 × 10^6^) were combined in serum-free GMEM in a conical tube, pelleted and the supernatant was aspirated. The pellet was broken by gentle tapping, and 1 ml of 50% (w/v) PEG 1500 (Roche Diagnostics, Basel, Switzerland), prewarmed at 37 °C, was gently added. The cells were incubated in the 50% PEG solution for 1 min with occasional stirring. Then, 1 ml of medium was added over a period of 1 min. Subsequently, an additional 3 ml of medium was added, the cells were spun down and the supernatant was discarded. The pellet was resuspended in complete ES cell medium and plated. Selection was applied after 48 h using hygromycin (200 mg/ml) and neomycin (100 mg/ml). Ten days after drug selection, ES cell-like colonies were picked and expanded under standard conditions. Immediately after selection, ESC-adult cancer cell hybrids, including EHe and EB colonies, had three different phenotypes: flat and not compact (80–85% in EHe and <30% in EB), fibroblast-like (15% in EHe and >70% in EB) and ESC-like (1–5% in EHe and 1% in EB) (data not shown). However, only the colonies with ESC-like shapes survived beyond the second passage, whereas the rest underwent cell cycle arrest and death. All the ES cell-like colonies were derived from individual fusion hybrids, which were individually picked under standard conditions. Upon serial passaging, all surviving colonies became uniform in appearance and had growth rates similar to ESCs.

### Karyotype analysis

A 25-cm flask at 60% cell confluence was treated with 0.04–0.1 *μ*g/ml colchicine for 3 h. Cells were recovered by trypsinization and treated with hypotonic (0.56% (w/v)) KCl solution for 15 min. The cells were centrifuged at 500 r.p.m., fixed by washing three times in fresh fixative (3 : 1 methanol : acetic acid) and dropped onto clean glass slides. The slides were air dried, stained with 4', 6-diamidino-2-phenylindole (DAPI) and observed under a microscope.

### FACS analysis

For analysis of DNA content, cells in a 10-cm dish were trypsinized, washed in PBS and fixed with 70% ethanol at 4 °C for 30 min. Next, RNase A was added to 500 *μ*l PBS at a final concentration of 20 *μ*g/ml, and the cells were incubated in this solution at 37 °C for 30 min. The cells were centrifuged at 1500 r.p.m. for 5 min, and the supernatant was discarded. Next, propidium iodide (PI) was added to 500 *μ*l of PBS at a final concentration of 50 *μ*g/ml, and the cells were incubated in this solution in the dark at room temperature for 30 min. Next, the cells were centrifuged at 1500 r.p.m. for 5 min, the supernatant was discarded and the cells were resuspended in 0.5 ml of PBS. Finally, the stained cells were analyzed with FACSCalibur (Becton, Dickinson and Company, Shanghai, China).

### RNA extraction, cDNA synthesis and qPCR

For real-time quantification of gene expression, RFP and GFP double-positive cells were sorted by FACS directly into RLT buffer (Qiagen GmbH, Shanghai, China), and the RNA was extracted using RNeasy microcolumns (Qiagen GmbH) according to the manufacturer's instructions. Random hexamer-primed first-strand cDNA was prepared with a SuperScript III Reverse Transcriptase Kit (cat. no. 18080-051; Invitrogen) according to the manufacturer's instructions. Real-time PCR was performed using the Bio-Rad MiniOpticon Real-Time PCR System (Bio-Rad, Shanghai, China) in a two-step RT-PCR. All RNA samples were treated with DNAse I (Takara, Dalian, China) to remove genomic DNA contamination. cDNA was synthesized with M-MLV reverse transcriptase (Promega, Madison, WI, USA) according to the instructions in the manual. Mouse-specific sequences for PCR primers were designed to generate amplicons of 150–250 bp required for real-time PCR detection using iQ SYBR Green Supermix (Bio-Rad). The mRNA abundances were determined by normalization of the data to the expression levels of glyceraldehyde-3-phosphate dehydrogenase mRNA. The primers used for PCR were in Supplementary Material.

### Bisulfite sequence analysis

Bisulfite treatment was performed with the EpiTect Bisulfite Kit (Qiagen, Shanghai, China; cat. no. 59104) according to the manufacturer's recommendations. For bisulfite sequencing of the mouse Oct4 promoter, primers were designed: outside forward, 5′-GAGGATTGGAGGTGTAATGGTTGTT-3′ outside reverse, 5′-CAAGCTTTGGGTTGAAATATTGGGTTTATTT-3′; inside forward, 5′-CAAGCTTTGGGTTGAAATATTGGGTTTATTT-3′; inside reverse, 5′-CGGATCCCTAAAACCAAATATCCAACCATA-3′. Amplified products were cloned into pCR2.1 -TOPO (Invitrogen). Ten randomly selected clones were sequenced with M13 forward and reverse primers.

### RNA-FISH

For RNA-FISH, a 559 bp RNA-FISH probe for nascent Oct4 transcripts was derived with the primers 5′-ATGGCTGGACACCTGGCTT-3′ and 5′-GCCTTCGCTCAGTTTCTCAT-3′, which were designed from Primer Premier 5 and Oligo 6. Then, another PCR was performed by adding the T7 promoter primer to the sense primer and the SP6 primer to the antisense primer from the previous reaction. The PCR product was purified with the Mini-DNA fragment Rapid Purification Kit (BioDev, Shanghai, China) and 1 *μ*g of the product was labeled by *in vitro* transcription with the Biotin-dUTP RNA Labeling Kit (Roche). The RNA product from the SP6 promoter was used as the probe for Oct4 and that from T7 was used as a control. The product was purified with Quick Spin RNA Columns (Qiagen). For each slide, 50 ng DNA probe, 5 *μ*g salmon sperm DNA and 20 *μ*g tRNA were used. Two volumes of 100% EtOH were added. After being dried, the samples were resuspended in 10 *μ*l hybridization mix and 1* μ*l RNA guard and left to dissolve at 37 °C and then denature at 80 °C for 10 min. Following overnight hybridization at 37 °C, slides were washed three times with 50% formamide/2 × standard saline citrate (SSC) at 45 °C for 5 min each, followed by three washes with 1 × SSC (prewarmed to 60 °C) at 45 °C for 5 min each. Detection was then performed using the TSA Kit (Molecular Probes, Shanghai, China; cat. no. T20931) was then performed. Cells were counterstained with DAPI, mounted in antifade (Southern Biotech, Shanghai, China; cat. no. 0100-20), viewed on an OLYMPUS IX51 (OLYMPUS, Shanghai, China) and analyzed with Image Pro Plus 5.1 (MediaCybernetics, Shanghai, China).

### cDNA microarray

Total RNA from ES cells, cancer cells, hybrid cells and differentiated hybrid cells (D7 and D14) were labeled with Cy5. Samples were hybridized to a Mouse Oligo Microarray (G4121B; Agilent, Shanghai, China) according to the manufacturer's protocol. Arrays were scanned with a G2565BA Microarray Scanner System (Agilent). Data were analyzed using GeneSpring GX software (Agilent). Microarray data have been deposited in the Gene Expression Omnibus database (GSE30965).

### *In vivo* analysis

For teratomas from ES cells, ES-cancer cell hybrids and cancer cells, ~1 × 10^6^ cells of each line were subcutaneously injected into the inguinal region of immunodeficient nude mice. The animal protocols were carried out in agreement with SIBS Guide for the Care and Use of Laboratory Animals and approved by Animal Care and Use Committee, Shanghai Institutes for Biological Sciences. The mice were monitored for tumor growth for 6 weeks. Then, teratomas or tumors were fixed with 4% PFA and embedded in paraffin. Sections were stained with hematoxylin and eosin.

### Immunohistochemistry

Teratomas were derived from ES cells, ES-lymphocytes, F9, P19, Hepa1-6, B16, EF, EP, ES-Hepa1-6 (EHe) and EB cells. Approximately 1 × 10^6^ cells of each clone were subcutaneously injected into the inguinal region of immunodeficient mice, and teratoma formation was assessed after 4–5 weeks of injection. Teratomas were fixed with 4% PFA, embedded in paraffin and stained with hematoxylin and eosin.

### *In vitro* differentiation

ES cells, EC cells and hybrids were harvested by treating the cells with 0.25% trypsin. The clumps of cells were transferred to a poly(2-hydroxyethyl methacrylate)-coated dish with DMEM/F12 supplemented with 20% KSR (Invitrogen), 2 mM l-glutamine, 1 × 10^−4^ nonessential amino acids, 1 × 10^−4^ M2-mercaptoethanol (Invitrogen) and 1% penicillin/streptomycin. The medium was changed every other day, and the EBs were harvested at days 3, 5, 7 and 9 to examine gene expression.

### Construction of shRNA plasmids, production of retroviral particles and infection of ES cells

shRNA oligos were designed by BLOCK-iT (https://rnaidesigner.invitrogen.com/rnaiexpress/). Each pair of oligos were annealed and ligated with the retroviral RNAi vector pSIREN-RetroQ (Clontech, Shanghai, China) precut with restriction enzymes. Plasmids were extracted with Maxi-prep Kit (Qiagen). After verifying the insert by sequencing, shRNA plasmids were co-transfected with helper plasmids into HEK 293 cells to produce the retrovirus. For infection, ES and EHe cells were incubated with the harvested retrovirus and centrifuged for 1.5 h at 30 °C. Then, cells were replenished with fresh medium. Puromycin was added to the medium 24 h after infection for enrichment of stably integrated cells.

### Western blot

Cell lysates were prepared as described previously.^52^ The proteins were separated on sodium dodecyl sulfate-polyacrylamide gel electrophoresis and transferred onto polyvinylidene difluoride membranes (Immobilon P; Millipore, Bedford, MA, USA). Blots were incubated with polyclonal antibodies against Foxa1 (1:500; Abcam, Shanghai, China) and actin (1:3000; Sigma, Shanghai, China), and then incubated with the appropriate horseradish peroxidase-conjugated secondary antibody (1:1000; Santa Cruz Biotechnology, Shanghai, China). Immunoreactive signals were detected by the use of an Immobilon Western Detection reagent (Millipore). Protein concentrations were measured with a Pierce BCA Protein Assay Kit (Thermo Fisher Scientific, Shanghai, China).

## Figures and Tables

**Table 1:** Tumors derived from ESC, cancer cell lines and hybrids

**Cell type**	**Latency weeks**	**Degree of differentiation**	**Types of tissues seen**
ESC	3–4	+++	Broad range of epithelial and mesodermal tissues
Lymphocyte	–	–	–
P19	1–2	+	Immature neuroepithelium, glandular epithelium, connective, adipose and cartilage
F9	1–2	0	Homogeneous undifferentiated
Hepa1-6	4–5	0	Homogeneous undifferentiated
B16	4	0	Homogeneous undifferentiated
EL hybrids	3–4	+++	Broad range of epithelial and mesodermal tissues
EP hybrids	1–2	+	Immature neuroepithelium, glandular epithelium, connective, adipose and cartilage
EF hybrids	1–2	0	Homogeneous undifferentiated
EHe hybrids	1–2	+	Mature adipose, and cartilage glandular epithelium, neuroepithelium and mesodermal tissues Undifferentiated hepatocarcinoma
EB hybrids	1–2	+	Mature adipose, and cartilage glandular epithelium, neuroepithelium and mesodermal tissues Undifferentiated hepatocarcinoma

Abbreviations: EB, ES-B16; EF, ES-F9; EHe, ES-Hepa1-6; EP, ES-P19; ESC, embryonic stem cells.

Degree of differentiation was determined by the percentage of undifferentiated cells in tumors. 0, 100% +, >50% ++, 20-50% +++, <20%

## Abbreviations

ES: embryonic stem
HCC: hepatocellular carcinoma
RNA-FISH: RNA-fluorescence *in situ* hybridization
DAPI: 4', 6-diamidino-2-phenylindole
DE: definitive endoderm
EC: embryonal cancer
EP: ES-P19
EF: ES-F9
EHe: ES-Hepa1-6
EB: ES-B16

## References

[bib1] Egger G, Liang G, Aparicio A, Jones PA. Epigenetics in human disease and prospects for epigenetic therapy. Nature 2004; 429: 457–463. 15164071 10.1038/nature02625

[bib2] Jones PA, Baylin SB. The fundamental role of epigenetic events in cancer. Nat Rev 2002; 3: 415–428. 10.1038/nrg816 12042769

[bib3] Hahn WC, Weinberg RA. Rules for making human tumor cells. N Engl J Med 2002; 347: 1593–1603. 12432047 10.1056/NEJMra021902

[bib4] Dougherty GJ, Dougherty ST. Exploiting the tumor microenvironment in the development of targeted cancer gene therapy. Cancer Gene Ther 2009; 16: 279–290. 18818709 10.1038/cgt.2008.72

[bib5] Topczewska JM, Postovit LM, Margaryan NV, Sam A, Hess AR, Wheaton WW et al. Embryonic and tumorigenic pathways converge via Nodal signaling: role in melanoma aggressiveness. Nat Med 2006; 12: 925–932. 16892036 10.1038/nm1448

[bib6] Esteller M. Epigenetics in cancer. N Engl J Med 2008; 358: 1148–1159. 18337604 10.1056/NEJMra072067

[bib7] Hochedlinger K, Blelloch R, Brennan C, Yamada Y, Kim M, Chin L et al. Reprogramming of a melanoma genome by nuclear transplantation. Genes Dev 2004; 18: 1875–1885. 15289459 10.1101/gad.1213504 PMC517407

[bib8] Blelloch RH, Hochedlinger K, Yamada Y, Brennan C, Kim M, Mintz B et al. Nuclear cloning of embryonal carcinoma cells. Proc Natl Acad Sci USA 2004; 101: 13985–13990. 15306687 10.1073/pnas.0405015101 PMC521109

[bib9] Li L, Connelly MC, Wetmore C, Curran T, Morgan JI. Mouse embryos cloned from brain tumors. Cancer Res 2003; 63: 2733–2736. 12782575

[bib10] Humpherys D, Eggan K, Akutsu H, Friedman A, Hochedlinger K, Yanagimachi R et al. Abnormal gene expression in cloned mice derived from embryonic stem cell and cumulus cell nuclei. Proc Natl Acad Sci USA 2002; 99: 12889–12894. 12235366 10.1073/pnas.192433399 PMC130555

[bib11] Humpherys D, Eggan K, Akutsu H, Hochedlinger K, Rideout WM III, Biniszkiewicz D et al. Epigenetic instability in ES cells and cloned mice. Science (New York, NY) 2001; 293: 95–97. 10.1126/science.1061402 11441181

[bib12] Carette JE, Pruszak J, Varadarajan M, Blomen VA, Gokhale S, Camargo FD et al. Generation of iPSCs from cultured human malignant cells. Blood 2010; 115: 4039–4042. 20233975 10.1182/blood-2009-07-231845 PMC2875096

[bib13] Kumano K, Arai S, Hosoi M, Taoka K, Takayama N, Otsu M et al. Generation of induced pluripotent stem cells from primary chronic myelogenous leukemia patient samples. Blood 2012; 119: 6234–6242. 22592606 10.1182/blood-2011-07-367441

[bib14] Miyoshi N, Ishii H, Nagai K, Hoshino H, Mimori K, Tanaka F et al. Defined factors induce reprogramming of gastrointestinal cancer cells. Proc Natl Acad Sci USA 2010; 107: 40–45. 20018687 10.1073/pnas.0912407107 PMC2806714

[bib15] Utikal J, Maherali N, Kulalert W, Hochedlinger K. Sox2 is dispensable for the reprogramming of melanocytes and melanoma cells into induced pluripotent stem cells. J Cell Sci 2009; 122(Part 19): 3502–3510. 19723802 10.1242/jcs.054783 PMC2746132

[bib16] Ramos-Mejia V, Fraga MF, Menendez P. iPSCs from cancer cells: challenges and opportunities. Trends Mol Med 2012; 18: 245–247. 22521522 10.1016/j.molmed.2012.04.001

[bib17] Zhang X, Cruz FD, Terry M, Remotti F, Matushansky I. Terminal differentiation and loss of tumorigenicity of human cancers via pluripotency-based reprogramming. Oncogene 2013; 32: 2249–2260 e1–e21. 22777357 10.1038/onc.2012.237 PMC3470785

[bib18] Kim J, Zaret KS. Reprogramming of human cancer cells to pluripotency for models of cancer progression. EMBO J 2015; 34: 739–747. 25712212 10.15252/embj.201490736 PMC4369311

[bib19] Kim K, Doi A, Wen B, Ng K, Zhao R, Cahan P et al. Epigenetic memory in induced pluripotent stem cells. Nature 2010; 467: 285–290. 20644535 10.1038/nature09342 PMC3150836

[bib20] Bar-Nur O, Russ HA, Efrat S, Benvenisty N. Epigenetic memory and preferential lineage-specific differentiation in induced pluripotent stem cells derived from human pancreatic islet beta cells. Cell Stem Cell 2011; 9: 17–23. 21726830 10.1016/j.stem.2011.06.007

[bib21] Tada M, Takahama Y, Abe K, Nakatsuji N, Tada T. Nuclear reprogramming of somatic cells by *in vitro* hybridization with ES cells. Curr Biol 2001; 11: 1553–1558. 11591326 10.1016/s0960-9822(01)00459-6

[bib22] Matveeva NM, Shilov AG, Kaftanovskaya EM, Maximovsky LP, Zhelezova AI, Golubitsa AN et al. *In vitro* and *in vivo* study of pluripotency in intraspecific hybrid cells obtained by fusion of murine embryonic stem cells with splenocytes. Mol Reprod Dev 1998; 50: 128–138. 9590528 10.1002/(SICI)1098-2795(199806)50:2<128::AID-MRD2>3.0.CO;2-M

[bib23] Cowan CA, Atienza J, Melton DA, Eggan K. Nuclear reprogramming of somatic cells after fusion with human embryonic stem cells. Science 2005; 309: 1369–1373. 16123299 10.1126/science.1116447

[bib24] Kimura H, Tada M, Nakatsuji N, Tada T. Histone code modifications on pluripotential nuclei of reprogrammed somatic cells. Mol Cell Biol 2004; 24: 5710–5720. 15216876 10.1128/MCB.24.13.5710-5720.2004 PMC480906

[bib25] Miller SC, Pavlath GK, Blakely BT, Blau HM. Muscle cell components dictate hepatocyte gene expression and the distribution of the Golgi apparatus in heterokaryons. Genes Dev 1988; 2: 330–340. 3378703 10.1101/gad.2.3.330

[bib26] Do JT, Han DW, Scholer HR. Reprogramming somatic gene activity by fusion with pluripotent cells. Stem Cell Rev 2006; 2: 257–264. 17848712 10.1007/BF02698052

[bib27] Do JT, Scholer HR. Nuclei of embryonic stem cells reprogram somatic cells. Stem Cells 2004; 22: 941–949. 15536185 10.1634/stemcells.22-6-941

[bib28] Do JT, Scholer HR. Comparison of neurosphere cells with cumulus cells after fusion with embryonic stem cells: reprogramming potential. Reprod Fertil Dev 2005; 17: 143–149. 15745639 10.1071/rd04120

[bib29] Matsumura H, Tada M, Otsuji T, Yasuchika K, Nakatsuji N, Surani A et al. Targeted chromosome elimination from ES-somatic hybrid cells. Nat Methods 2007; 4: 23–25. 17086180 10.1038/nmeth973

[bib30] Saito Y, Hibino S, Saito H. Alterations of epigenetics and microRNA in hepatocellular carcinoma. Hepatol Res 2014; 44: 31–42. 23617364 10.1111/hepr.12147

[bib31] Martin M, Herceg Z. From hepatitis to hepatocellular carcinoma: a proposed model for cross-talk between inflammation and epigenetic mechanisms. Genome Med 2012; 4: 8. 22293089 10.1186/gm307 PMC3334556

[bib32] Matsuda Y, Wakai T, Kubota M, Takamura M, Yamagiwa S, Aoyagi Y et al. Clinical significance of cell cycle inhibitors in hepatocellular carcinoma. Med Mol Morphol 2013; 46: 185–192. 23640750 10.1007/s00795-013-0047-7

[bib33] Knappe N, Novak D, Weina K, Bernhardt M, Reith M, Larribere L et al. Directed de-differentiation using partial reprogramming induces invasive phenotype in melanoma cells. Stem Cells 2016; 34: 832–846. 26753613 10.1002/stem.2284

[bib34] Yao JY, Zhang L, Zhang X, He ZY, Ma Y, Hui LJ et al. H3K27 trimethylation is an early epigenetic event of p16INK4a silencing for regaining tumorigenesis in fusion reprogrammed hepatoma cells. J Biol Chem 2010; 285: 18828–18837. 20382980 10.1074/jbc.M109.077974 PMC2881805

[bib35] Peng S, Maihle NJ, Huang Y. Pluripotency factors Lin28 and Oct4 identify a sub-population of stem cell-like cells in ovarian cancer. Oncogene 29: 2153–2159. 10.1038/onc.2009.500 20101213

[bib36] Zaret KS, Carroll JS. Pioneer transcription factors: establishing competence for gene expression. Genes Dev 2011; 25: 2227–2241. 22056668 10.1101/gad.176826.111 PMC3219227

[bib37] Xu C, Lu X, Chen EZ, He Z, Uyunbilig B, Li G et al. Genome-wide roles of Foxa2 in directing liver specification. J Mol Cell Biol 2012; 4: 420–422. 22750789 10.1093/jmcb/mjs037 PMC3612007

[bib38] Li Z, Tuteja G, Schug J, Kaestner KH. Foxa1 and Foxa2 are essential for sexual dimorphism in liver cancer. Cell 2012; 148: 72–83. 22265403 10.1016/j.cell.2011.11.026 PMC3266536

[bib39] Silva J, Chambers I, Pollard S, Smith A. Nanog promotes transfer of pluripotency after cell fusion. Nature 2006; 441: 997–1001. 16791199 10.1038/nature04914

[bib40] Postovit LM, Margaryan NV, Seftor EA, Hendrix MJ. Role of nodal signaling and the microenvironment underlying melanoma plasticity. Pigment Cell Melanoma Res 2008; 21: 348–357. 18444961 10.1111/j.1755-148X.2008.00463.x

[bib41] Kasemeier-Kulesa JC, Teddy JM, Postovit LM, Seftor EA, Seftor RE, Hendrix MJ et al. Reprogramming multipotent tumor cells with the embryonic neural crest microenvironment. Dev Dyn 2008; 237: 2657–2666. 18629870 10.1002/dvdy.21613 PMC2570047

[bib42] Hendrix MJ, Seftor EA, Seftor RE, Kasemeier-Kulesa J, Kulesa PM, Postovit LM. Reprogramming metastatic tumour cells with embryonic microenvironments. Nat Rev 2007; 7: 246–255. 10.1038/nrc2108 17384580

[bib43] Kulesa PM, Kasemeier-Kulesa JC, Teddy JM, Margaryan NV, Seftor EA, Seftor RE et al. Reprogramming metastatic melanoma cells to assume a neural crest cell-like phenotype in an embryonic microenvironment. Proc Natl Acad Sci USA 2006; 103: 3752–3757. 16505384 10.1073/pnas.0506977103 PMC1450149

[bib44] Jaenisch R, Young R. Stem cells, the molecular circuitry of pluripotency and nuclear reprogramming. Cell 2008; 132: 567–582. 18295576 10.1016/j.cell.2008.01.015 PMC4142810

[bib45] Lin L, Miller CT, Contreras JI, Prescott MS, Dagenais SL, Wu R et al. The hepatocyte nuclear factor 3 alpha gene, HNF3alpha (FOXA1), on chromosome band 14q13 is amplified and overexpressed in esophageal and lung adenocarcinomas. Cancer Res 2002; 62: 5273–5279. 12234996

[bib46] Grasso CS, Wu YM, Robinson DR, Cao X, Dhanasekaran SM, Khan AP et al. The mutational landscape of lethal castration-resistant prostate cancer. Nature 2012; 487: 239–243. 22722839 10.1038/nature11125 PMC3396711

[bib47] Robbins CM, Tembe WA, Baker A, Sinari S, Moses TY, Beckstrom-Sternberg S et al. Copy number and targeted mutational analysis reveals novel somatic events in metastatic prostate tumors. Genome Res 2011; 21: 47–55. 21147910 10.1101/gr.107961.110 PMC3012925

[bib48] Cancer Genome Atlas N.. Comprehensive molecular portraits of human breast tumours. Nature 2012; 490: 61–70. 23000897 10.1038/nature11412 PMC3465532

[bib49] Wang R, Liang J, Jiang H, Qin LJ, Yang HT. Promoter-dependent EGFP expression during embryonic stem cell propagation and differentiation. Stem Cells Dev 2008; 17: 279–289. 18447643 10.1089/scd.2007.0084

[bib50] Privratsky JR, Tourdot BE, Newman DK, Newman PJ. The anti-inflammatory actions of platelet endothelial cell adhesion molecule-1 do not involve regulation of endothelial cell NF-kappa B. J Immunol 2010; 184: 3157–3163. 20173029 10.4049/jimmunol.0901944 PMC3628820

[bib51] LeComte MD, Shimada IS, Sherwin C, Spees JL. Notch1-STAT3-ETBR signaling axis controls reactive astrocyte proliferation after brain injury. Proc Natl Acad Sci USA 2015; 112: 8726–8731. 26124113 10.1073/pnas.1501029112 PMC4507218

[bib52] Fujimori K, Ueno T, Nagata N, Kashiwagi K, Aritake K, Amano F et al. Suppression of adipocyte differentiation by aldo-keto reductase 1B3 acting as prostaglandin F2alpha synthase. J Biol Chem 2010; 285: 8880–8886. 20093363 10.1074/jbc.M109.077164 PMC2838309

