# Supplementary figures and images for: Tumorigenic potential is restored during differentiation in fusion-reprogrammed cancer cells
Source: Cell Death Dis. 2016 Jul 28;7(7):e2314–. doi: 10.1038/cddis.2016.189 (PMC4973342; doi:10.1038/cddis.2016.189)

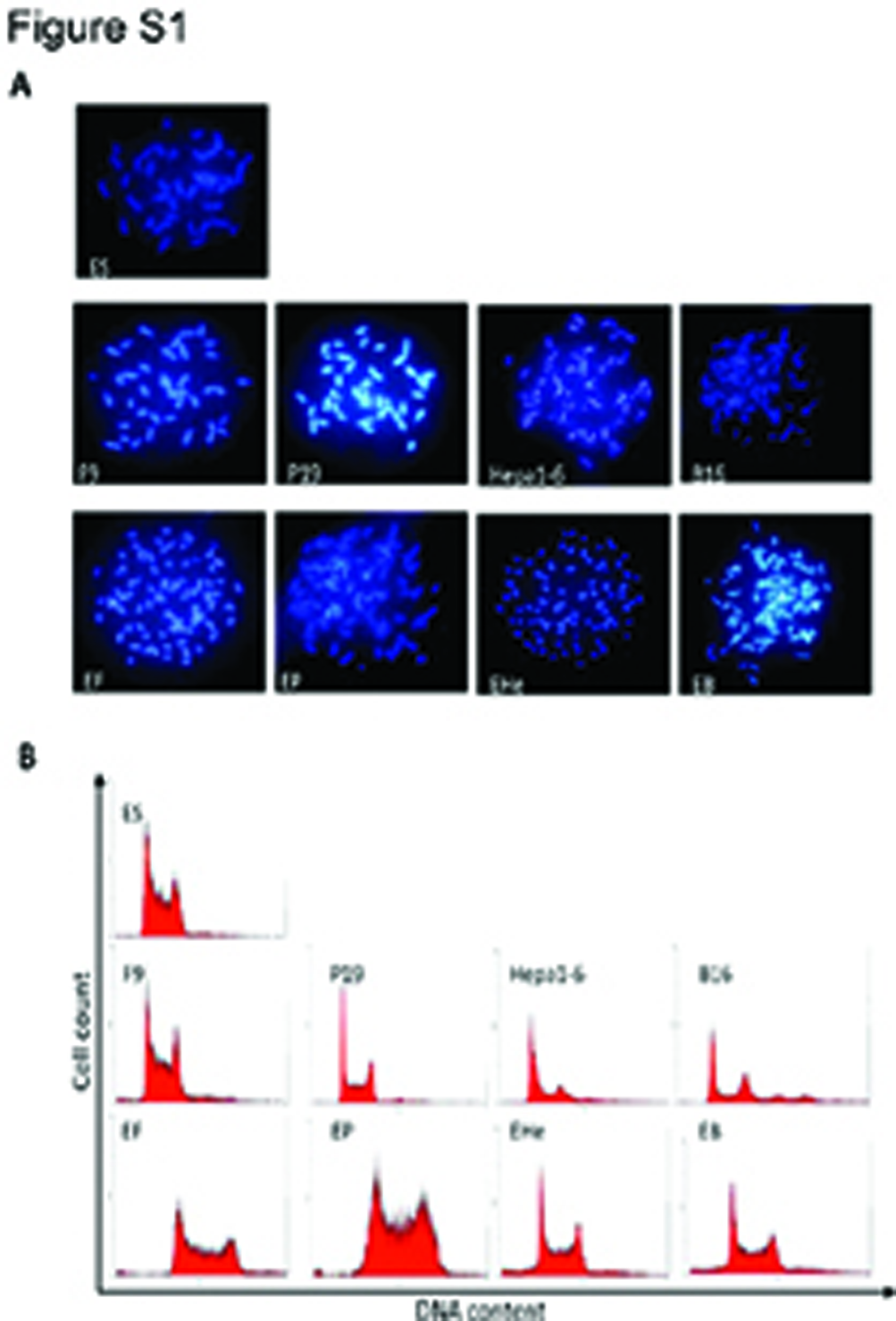

Supplement: Supplementary Figure S1 [file cddis2016189x1.tif]

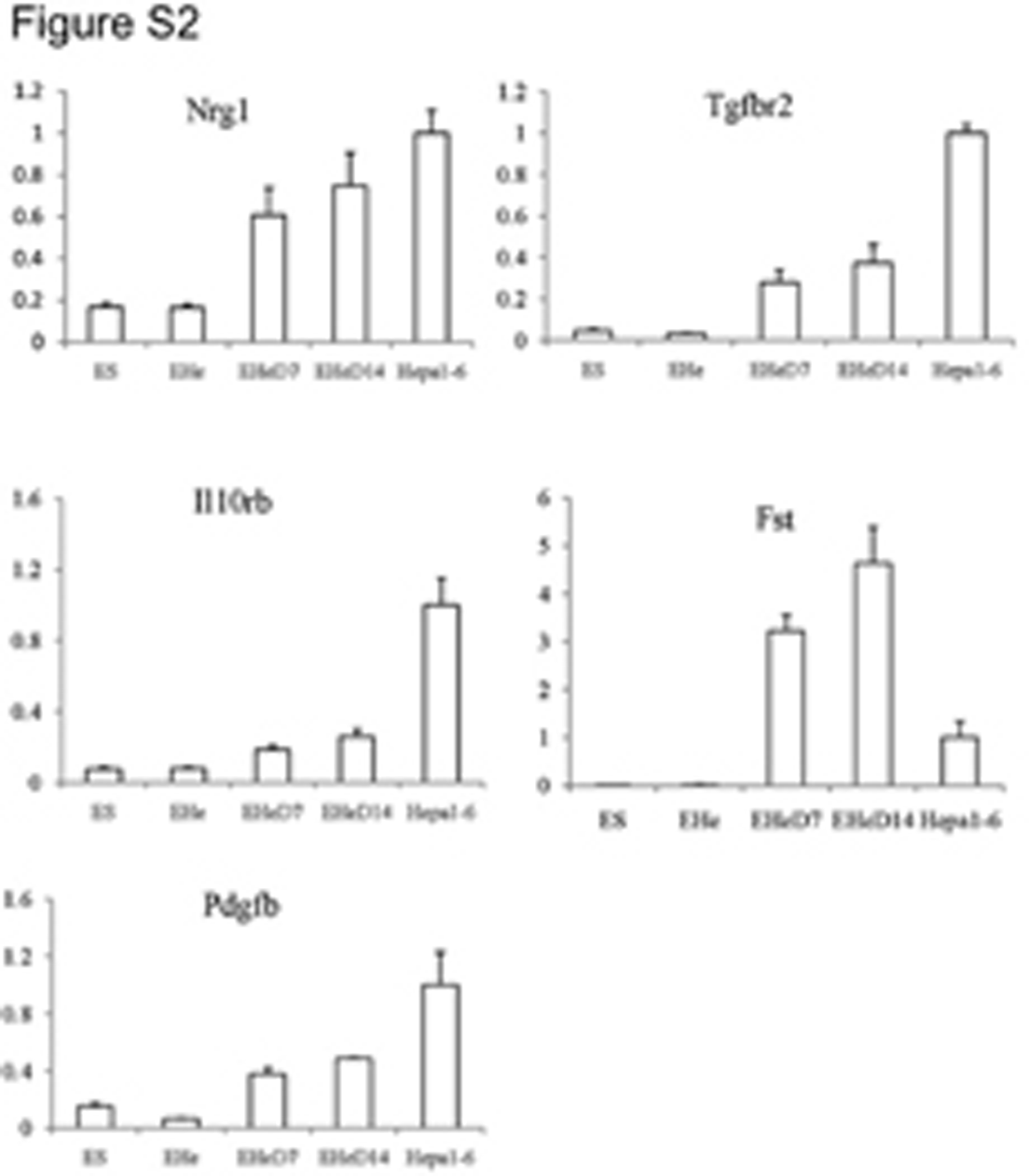

Supplement: Supplementary Figure S2 [file cddis2016189x2.tif]

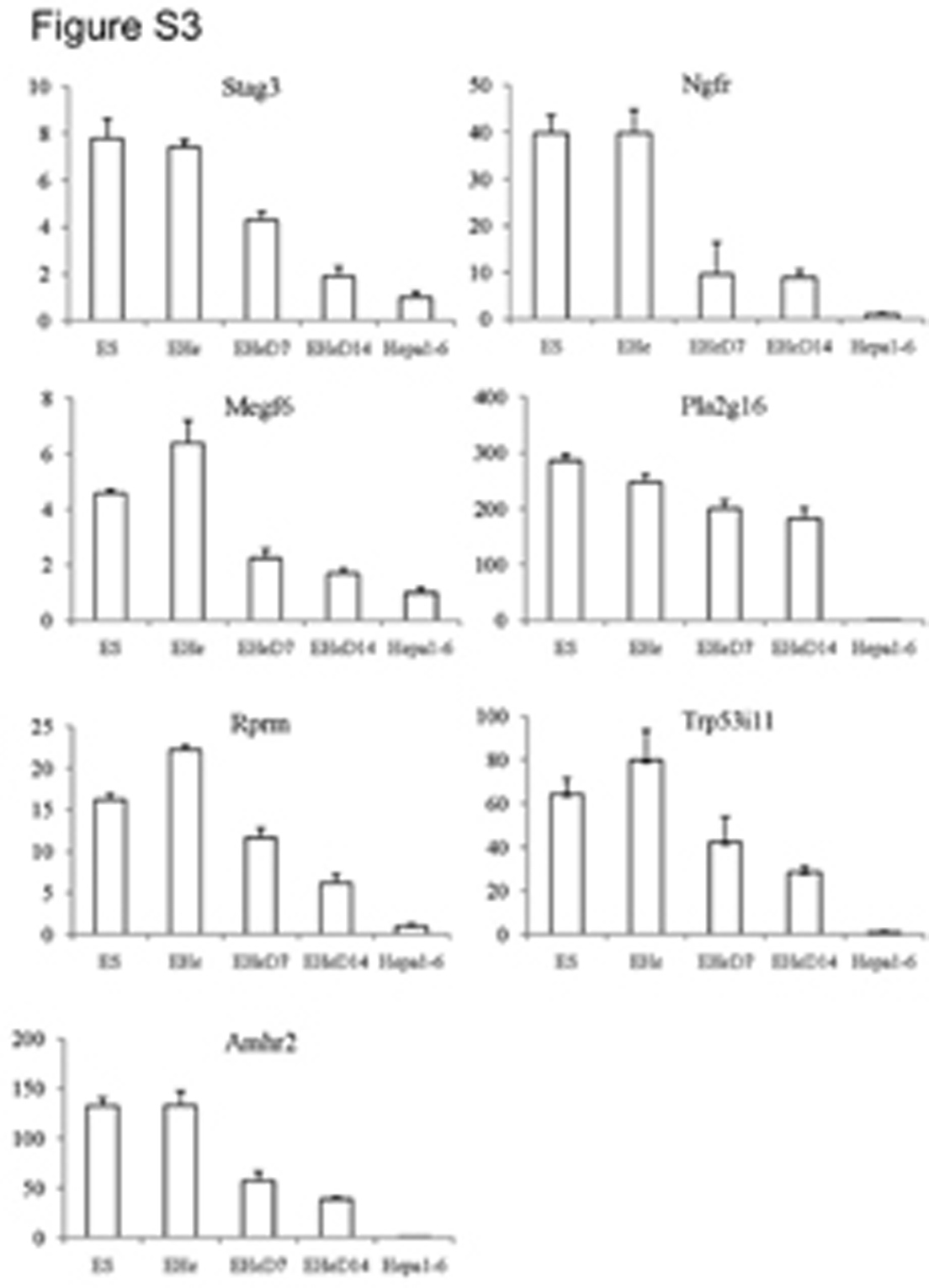

Supplement: Supplementary Figure S3 [file cddis2016189x3.tif]
