# Supplementary material for: Tumorigenic potential is restored during differentiation in fusion-reprogrammed cancer cells
Source: Cell Death Dis. 2016 Jul 28;7(7):e2314–. doi: 10.1038/cddis.2016.189 (PMC4973342; doi:10.1038/cddis.2016.189)
Supplement: Supplementary Data 1 [file cddis2016189x5.doc]

**SUPPLEMENTAL DATA:**

Table 1. Tumor candidates

| mouse | embryonic | P19 | teratocarcinoma | McBurney MW , et al. Control of muscle and neuronal differentiation in a cultured embryonal carcinoma cell line. Nature 299: 165-167, 1982. PubMed: 7110336 |
| --- | --- | --- | --- | --- |
|  |  | F9 | testicular teratoma | Strickland S , Mahdavi V . The induction of differentiation in teratocarcinoma stem cells by retinoic acid. Cell 15: 393-403, 1978. PubMed: 214238 |
|  | adult | B16 | melanoma | Fidler IJ . Biological behavior of malignant melanoma cells correlated to their survival in vivo. Cancer Res. 35: 218-224, 1975. PubMed: 1109790 |
|  |  | Hepa1-6 | hepatoma | Darlington GJ , et al. Expression of liver phenotypes in cultured mouse hepatoma cells. J. Natl. Cancer Inst. 64: 809-819, 1980. PubMed: 6102619 |
