# Supplementary material for: Tumorigenic potential is restored during differentiation in fusion-reprogrammed cancer cells
Source: Cell Death Dis. 2016 Jul 28;7(7):e2314–. doi: 10.1038/cddis.2016.189 (PMC4973342; doi:10.1038/cddis.2016.189)
Supplement: Supplementary Data 2 [file cddis2016189x6.doc]

**SUPPLEMENTAL DATA:**

| **Table 2. Genome-wide gene expression profile analysis of ES-Hepa hybrids**   1. **Cell Cycle** | | | | | | |
| --- | --- | --- | --- | --- | --- | --- |
| **Gene Symbol** | | **[ES]/[Hepa]** | **[EHe]/[Hepa]** | **[EHeD7]/[Hepa** | **[EHeD14]/[Hepa]** |  |
| Ccnd1 | 0.173 | | 0.11 | 0.096 | 0.048 |  |
| Bub1b | 7.333 | | 8.084 | 7.345 | 7.134 |  |
| Plk2 | 0.133 | | 0.076 | 0.289 | 0.264 |  |
| Cdk6 | 0.14 | | 0.019 | 0.105 | 0.164 |  |
| Atmin | 4.982 | | 7.698 | 4.778 | 4.197 |  |
| Anapc10 | 4.149 | | 4.699 | 3.507 | 3.953 |  |
| Stag3 | 649.723 | | 411.467 | 218.32 | 105.44 |  |
| Espl1 | 9.875 | | 6.095 | 7.204 | 6.209 |  |

1. Cytokine cytokine receptor interaction

| **Gene Symbol** | | **[ES]/[Hepa]** | **EHe]/[Hepa]** | **[EHeD7]/[Hepa]** | **[EHeD14]/[Hepa]** |
| --- | --- | --- | --- | --- | --- |
| Tgfb1 | 0.077 | | 0.043 | 0.223 | 0.257 |
| Il10rb | 0.075 | | 0.039 | 0.209 | 0.197 |
| Egfr | 0.021 | | 0.061 | 0.173 | 0.112 |
| Il17rc | 0.21 | | 0.267 | 0.272 | 0.325 |
| Met | 0.009 | | 0.009 | 0.031 | 0.035 |
| Ccl7 | 0.079 | | 0.155 | 0.064 | 0.172 |
| Pdgfc | 0.087 | | 0.235 | 0.168 | 0.17 |
| Flt3l | 0.112 | | 0.167 | 0.146 | 0.23 |
| Tgfbr2 | 0.086 | | 0.126 | 0.155 | 0.117 |
| Pdgfb | 0.068 | | 0.046 | 0.24 | 0.234 |
| Ifngr1 | 0.036 | | 0.061 | 0.086 | 0.101 |

**C.** Apoptosis

| **Gene Symbol** | | **[ES]/[Hepa]** | | **EHe]/[Hepa]** | | **[EHeD7]/[Hepa]** | | **[EHeD14]/[Hepa]** | |
| --- | --- | --- | --- | --- | --- | --- | --- | --- | --- |
| Il10rb | 0.075 | | 0.039 | | 0.209 | | 0.197 | |  |
| Tifa | 0.014 | | 0.011 | | 0.029 | | 0.03 | |  |
| Il13ra1 | 0.068 | | 0.085 | | 0.249 | | 0.247 | |  |
| Il17rc | 0.21 | | 0.267 | | 0.272 | | 0.325 | |  |
| Tnik | 30.129 | | 36.159 | | 7.144 | | 12.274 | |  |
| Ngfr | 37.031 | | 20.279 | | 7.423 | | 3.869 | |  |
| Capn2 | 0.053 | | 0.169 | | 0.291 | | 0.307 | |  |
| Bik | 61.077 | | 77.028 | | 43.976 | | 40.128 | |  |
| Ntrk1 | 25.673 | | 31.017 | | 8.002 | | 5.119 | |  |
| Nfkb1 | 0.119 | | 0.134 | | 0.259 | | 0.257 | |  |
| Akap2 | 0.174 | | 0.071 | | 0.218 | | 0.266 | |  |
| Nfkb2 | 0.058 | | 0.029 | | 0.094 | | 0.09 | |  |

1. TGF beta signaling pathway

| **Gene Symbol** | **[ES]/[Hepa]** | **EHe]/[Hepa]** | **[EHeD7]/[Hepa]** | **[EHeD14]/[Hepa]** |
| --- | --- | --- | --- | --- |
| Tgfb1 | 0.077 | 0.043 | 0.223 | 0.257 |
| Myc | 0.063 | 0.012 | 0.035 | 0.057 |
| Thbs1 | 0.002 | 0.002 | 0.06 | 0.051 |
| Amhr2 | 27.145 | 31.883 | 24.875 | 18.364 |
| Smad3 | 0.15 | 0.113 | 0.191 | 0.206 |
| Bmpr2 | 0.026 | 0.016 | 0.021 | 0.024 |
| Tgfbr2 | 0.086 | 0.126 | 0.155 | 0.117 |

1. Esrb signaling pathway

| **Gene Symbol** | **[ES]/[Hepa]** | **EHe]/[Hepa]** | **[EHeD7]/[Hepa]** | **[EHeD14]/[Hepa]** |
| --- | --- | --- | --- | --- |
| Tgfa | 0.032 | 0.028 | 0.079 | 0.095 |
| Camk2d | 0.018 | 0.02 | 0.099 | 0.159 |
| Nrg1 | 0.062 | 0.037 | 0.106 | 0.16 |
| Stat5b | 0.046 | 0.096 | 0.161 | 0.228 |
| Camk2d | 0.14 | 0.168 | 0.232 | 0.313 |
| Megf6 | 33.862 | 30.617 | 22.519 | 15.46 |
| Nrg1 | 0.06 | 0.041 | 0.112 | 0.179 |

1. **MAPK signaling pathway**

| **Gene Symbol** | **[ES]/[Hepa]** | **EHe]/[Hepa]** | **[EHeD7]/[Hepa]** | **[EHeD14]/[Hepa]** |
| --- | --- | --- | --- | --- |
| Rac1 | 0.251 | 0.285 | 0.306 | 0.283 |
| Tgfb1 | 0.077 | 0.043 | 0.223 | 0.257 |
| Nfatc2 | 4.232 | 4.508 | 3.053 | 3.454 |
| Pla2g16 | 203.055 | 270.618 | 123.867 | 160.955 |
| Fos | 0.086 | 0.022 | 0.164 | 0.213 |
| Fgf3 | 10.585 | 5.568 | 9.506 | 7.017 |
| Ngfr | 37.031 | 20.279 | 7.423 | 3.869 |
| Megf6 | 33.862 | 30.617 | 22.519 | 15.46 |
| Tgfbr2 | 0.086 | 0.126 | 0.155 | 0.117 |
| Dusp6 | 0.115 | 0.157 | 0.185 | 0.218 |
| Rps6ka4 | 0.115 | 0.248 | 0.215 | 0.222 |
| Tmeff1 | 714.609 | 837.321 | 787.691 | 697.041 |
| Myc | 0.093 | 0.053 | 0.068 | 0.078 |
| Junb | 0.232 | 0.151 | 0.21 | 0.199 |

1. p53_signaling_pathway

| **Gene Symbol** | **[ES]/[Hepa]** | **EHe]/[Hepa]** | **[EHeD7]/[Hepa]** | **[EHeD14]/[Hepa]** |
| --- | --- | --- | --- | --- |
| Thbs1 | 0.028 | 0.03 | 0.059 | 0.04 |
| Serpine1 | 0.196 | 0.209 | 0.196 | 0.201 |
| Thbs1 | 0.002 | 0.002 | 0.06 | 0.051 |
| Rbak | 9.044 | 7.094 | 3.992 | 4.233 |
| Thbs1 | 0.014 | 0.014 | 0.077 | 0.06 |
| Bai1 | 3.971 | 8.063 | 5.164 | 3.834 |
| Rprm | 37.347 | 109.425 | 53.167 | 47.687 |
| Chek2 | 5.345 | 3.919 | 4.194 | 4.525 |
| Trp53i11 | 43.286 | 64.527 | 38.521 | 26.638 |
