# Supplementary material for: Tumorigenic potential is restored during differentiation in fusion-reprogrammed cancer cells
Source: Cell Death Dis. 2016 Jul 28;7(7):e2314–. doi: 10.1038/cddis.2016.189 (PMC4973342; doi:10.1038/cddis.2016.189)
Supplement: Supplementary Materials [file cddis2016189x7.doc]

**SUPPLEMENTAL MATERIALS AND METHODS**

Primers for RT-PCR and Q-PCR

| Name | Primers | Application |
| --- | --- | --- |
| Oct4 sense | AGGCCCGGAAGAGAAAGCGAACTA | RT-PCR |
| Oct4 antisense | TGGGGGCAGAGGAAAGGATACAGC | RT-PCR |
| Rex1 sense | GAAGCACATGCTTGTCCACGG | RT-PCR |
| Rex1 antisense | GCCTTTGCGTGGGTTAGGATG | RT-PCR |
| β-actin sense | GGTGGGAATGGGTCAGAAGG | RT-PCR |
| β-actin antisense | AGGAAGAGGATGCGCCAGTG | RT-PCR |
| Sox2 sense | CGACCGGCGGCAACCAGAAGAACA | RT-PCR |
| Sox2 antisense | GCCGGCGCCCACCCCAACC | RT-PCR |
| Nanog sense | AAGTACCTCAGCCTCCAGCAGATG | RT-PCR |
| Nanog antisense | AGAAAGTCCTCCCCGAAGTTATGG | RT-PCR |
| Tyrosinase sense | CTGGCAGATCATTTGTAGCA | RT-PCR |
| Tyrosinase antisense | AGGCATTGTGCATGCTGCTT | RT-PCR |
| Trp-1 sense | CCTGGCCAAGAAGAGTATCC | RT-PCR |
| Trp-1 antisense | CACGTCACACTCGTTCTTCC | RT-PCR |
| Alb sense | GCTACGGCACAGTGCTTG | RT-PCR |
| Alb antisense | CAGGATTGCAGACAGATAGTC | RT-PCR |
| Ttr sense | CTCACCACAGATGAGAAG | RT-PCR |
| Ttr antisense | GGCTGAGTCTCTCAATTC | RT-PCR |
| p19ARFsense | ATGGGTCGCAGGTTCTTGGTCACTG | RT-PCR |
| p19ARFantisense | CTAGACACGCTAGCATCGCTAGAAG | RT-PCR |
| p16 Ink4a sense | ATGGAGTCCGCTGCAGACAGA | RT-PCR |
| p16 Ink4a antisense | GTGCTTGAGCTGAAGCTA | RT-PCR |
| C-fos sense | GGTCATCGGGGATCTTGC | RT-PCR |
| C-fos antisense | ATGGGCTCTCCTGTCAAC | RT-PCR |
| C-jun sense | GATGGAAACGACCTTCTACGAC | RT-PCR |
| C-jun antisense | ACGTTCTTGGGGCACAAGAACT | RT-PCR |
| Bcl2 sense | AGAACAGGGTATGATAACCGGG | RT-PCR |
| Bcl2 antisense | ACATGACCCCACCGAACTCAAA | RT-PCR |
| Stag3 Forward primer | TCACAGCAGAGGCCAGCCCT | Q-PCR |
| Stag3 Reverse primer | AGGGGTTGTGGGGCCACTGT | Q-PCR |
| Egfr Forward primer | CTGCCCACCACTCATGCTGTACAA | Q-PCR |
| Egfr Reverse primer | GGCCCACAGGCTCGGACAGATCT | Q-PCR |
| Tgfbr2 Forward primer | ACATCAGCTCCACGTGCGCC | Q-PCR |
| Tgfbr2 Reverse primer | GTAGACCTCGGCGAAGCGGC | Q-PCR |
| Pdgfb Forward primer | GTGACCCCTCGGCCTGTGACT | Q-PCR |
| Pdgfb Reverse primer | CCTTCAGGGCCGCCTTGTCTTA | Q-PCR |
| Il10rb Forward primer | GGCCACCCCCATCACAGCAC | Q-PCR |
| Il10rb Reverse primer | CAGACGGGGGCTCTGAGGCA | Q-PCR |
| Tnik Forward primer | AGGATCACCCGTGCTCCCCC | Q-PCR |
| Tnik Reverse primer | GCTTTCTTATAGCTAGCTGGCCGAC | Q-PCR |
| Ngfr Forward primer | CCAACCACGTGGACCCGTGC | Q-PCR |
| Ngfr Reverse primer | CGGCCAGGGATCTCCTCGCAT | Q-PCR |
| Capn2 Forward primer | ATGGAAGCGGCCCACGGAGA | Q-PCR |
| Capn2 Reverse primer | CAGCCAGCAGCCAGCAGTCC | Q-PCR |
| Ntrk1 Forward primer | GACTCCTGGCGCCTGGGTGT | Q-PCR |
| Ntrk1 Reverse primer | TGCCCCAGCTGCACTTCACT | Q-PCR |
| Tgfb1 Forward primer | CGAGGCGGTGCTCGCTTTGTA | Q-PCR |
| Tgfb1 Reverse primer | CATAGATGGCGTTGTTGCGGTCCA | Q-PCR |
| Fst Forward primer | TGCAACTCCATCTCGGAAGAAACGG | Q-PCR |
| Fst Reverse primer | GCCCAAAGGCTATGTCAACACTGA | Q-PCR |
| Amhr2 Forward primer | AGGTCATGCGCCATCGGGGA | Q-PCR |
| Amhr2 Reverse primer | GCTGGGTGCCAGCCTCCAAAAT | Q-PCR |
| Nrg1 Forward primer | TGGAGGCGAGTGCTTCATGGTG | Q-PCR |
| Nrg1 Reverse primer | CCCAAGATGCTTGTAGAAGCTGGCC | Q-PCR |
| Megf6 Forward primer | AGCTTCTGCGAGCACGCCTG | Q-PCR |
| Megf6 Reverse primer | AGCTGGGCAGATGCACGAGC | Q-PCR |
| Pla2g16 Forward primer | GCGGCCATTAAACCAGTCCCC | Q-PCR |
| Pla2g16 Reverse primer | AGCGTGGATGCCTTTGCTTGGG | Q-PCR |
| Rprm Forward primer | TGTTCGTCCCGCGCTACAGC | Q-PCR |
| Rprm Reverse primer | GCCACGTCGGTCTGGTTGCC | Q-PCR |
| Rbak Forward primer | CAGCGAACAGACTCGGCTGTGC | Q-PCR |
| Rbak Reverse primer | GACACTGCTGACTGACCGAGCAGG | Q-PCR |
| Trp53i11 Forward primer | GGGGCTCAGGGTCTGGCAGT | Q-PCR |
| Trp53i11 Reverse primer | CCGTAGAGGCGGATGGGGGT | Q-PCR |
