# Supplementary material for: Tumorigenic potential is restored during differentiation in fusion-reprogrammed cancer cells
Source: Cell Death Dis. 2016 Jul 28;7(7):e2314–. doi: 10.1038/cddis.2016.189 (PMC4973342; doi:10.1038/cddis.2016.189)
Supplement: Supplementary Figure Legends [file cddis2016189x8.doc]

**Figure S1.** Karyotype analysis of ES cells, cancer cells and ES-cancer cell hybrids. (A) Representative metaphase spread of hybrid cells and the fusion parental cells. (B) DNA ploidy. Parental ES cells, cancer cells and hybrid cells were stained with propidium iodide and subjected to FACS analysis. EP: ES-P19, EF: ES-F9, EHe: ES-Hepa1-6, EB: ES-B16

**Figure S2.** Real-time PCR confirmed downregulated mRNA expression of several genes corresponding to the array data. Error bars, S.E. of the average values.

**Figure S3.** Real-time PCR confirmed upregulated mRNA expression of several genes corresponding to the array data. Error bars, S.E. of the average values.
